# Relationship between anastomotic technique, incisional hernia, and quality of life—the Minimally Invasive Right Colectomy Anastomosis STudy (MIRCAST)

**DOI:** 10.1093/bjs/znaf250

**Published:** 2025-12-08

**Authors:** Marcos Gómez Ruiz, MIRCAST study group (collaborative), Juan García Cardo, Marcos Gómez Ruiz, Eloy Espin Basany, Mindaugas Tiskus, Ugo Pace, Tarja Pinta, Paolo Pietro Bianchi, Andrea Coratti, Roberto Persiani, Roberto Coppola, Nuria Truan Alonso, Franco Marinello, Miquel Kraft Carre, Mirjana Komljen, Shadi Andos, Paolo Delrio, Daniela Rega, Giuseppe Giuliani, Lucia Salvischiani, Alberto Biondi, Laura Lorenzon, Damiano Caputo, Vincenzo La Vaccara, Daniel Fernández Martinez, Carmen Cagigas Fernández, Lidia Cristobal Poch, Gina Lladó-Jordan, Camilo Palazuelos Calderón, Lucía Lavín Alconero, Vincenzo Vigorita, Raquel Sánchez Santos, Paula Fernández Rodriguez, Fernando Jimenez Escobar, Tamara Fernández Miguel, Vicente Portugal Porras, Alejandro Romero de Diego, Maria Ruíz Soriano, Beatriz de Andrés Asenjo, Dursun Bugra, Emre Balik, Emre Özoran, Andrea Muratore, Marcello Calabrò, Antonio La Terra, Ángel Reina Duarte, Pälvi Vento, Inna Lupina, David Moro-Valdezate, José Martín-Arévalo, Juan Ocaña Jiménez, Araceli Ballestero—Pérez, Ellen Van Eetvelde, Daniel Jacobs-Tulleneers-Thevissen, Irshad Shaikh, Dolly Dowsett, Esther Kreisler Moreno, Ana Gálvez Saldaña, Antonino Spinelli, Francesca Di Candido, Luis Miguel Jimenez Gomez, Elena Hurtado Caballero, Andreas Türler, Anna Krappitz, Luca Morelli, Annalisa Comandatore, Matteo Palmeri, Vicente Simó, Jorge Arredondo Chaves, Benno Mann, Gintautas Virakas, Jim Khan, Ismail Gögenur, Niclas Dohrn, Eduardo Ferrero Herrero, Eduardo Rubio González, Javier Sanchez Gonzalez, Ekta Choolani Bhojwani, Francesk Mulita, Vasileios Leivaditis, Goran Šantak, Matteo Frasson, Marta Nieto, Jakob Lykke, Niclas Dohrn, Mauro Garino, Chiara Marafante, Antonio Arroyo, Cristina Lillo-García, Carlos Placer Galan, José María Enriquez Navascués, Wanda Luisa Petz, Simona Borin, Philippe Rouanet, Christophe Taoum, Alain Valverde, Markus Winny, Çağrı Büyükkasap, Benoit Romain, Orestis Ioannidis, Giuseppe Spinoglio, David Jayne, Roger Gerjy, Sanjay Chaudhri, Luis Sánchez-Guillén, Alexis Ulrich, Tero Rautio, Jesus Bollo Rodriguez, Nuno Rama, Federico Perna, Eric Rullier, Fernando Mendoza, Thalia Petropoulou, Arto Turunen, Mauricio García Alonso, Anne Mattila, Julian Hance, Bertrand Trilling, Imma Prós Ribas, Adeline Germain, Kai Leong

**Affiliations:** Colorectal Surgery Unit, General Surgery Department, Marqués de Valdecilla University Hospital, Santander, 39008, Spain; Valdecilla Biomedical Research Institute (IDIVAL), Santander, 39011, Spain; Faculty of Medicine, University of Cantabria, Santander, 39011, Spain

## Introduction

Standard treatment for right colon cancer remains surgical resection in the vast majority of cases. This is often performed using a minimally invasive approach, because of the numerous advantages reported in RCTs^1–3^. Different anastomotic techniques are used after minimally invasive bowel resection, performed in either an extracorporeal (ECA) or intracorporeal (ICA) manner. Several publications including meta-analyses, large prospective cohort studies, RCTs^4–9^, and the previous Minimally Invasive Right Colectomy Anastomosis STudy (MIRCAST) report^10^ have shown potential advantages in the use of ICA when compared with ECA, specifically faster bowel recovery, lower overall postoperative complications, and fewer incisional hernias (IH).

It is still unclear why ICA is associated with faster bowel recovery or lower overall postoperative complications. Different explanations have been suggested, including less traction on the mesentery when performing ICA^11^, or better perfusion of the bowel in obese patients^12^. More robust evidence is available regarding the relationship between ECA and IH. Numerous comparative studies have been conducted comparing ICA and ECA, observing a higher rate of IH in patients with ECA and midline incisions. A Pfannenstiel incision, more frequently used for specimen extraction after ICA, is protective against IH formation^13^.

Although many publications during the last decades have addressed the impact of rectal cancer surgery on patients’ quality of life (QoL)^14–16^, there is a clear gap in knowledge regarding QoL after minimally invasive right colectomy, or the impact that anastomotic technique might have on QoL. Most of the publications in which this topic has been addressed included both colon and rectal cancer patients and analysed the outcomes of both groups together^17–18^. None of these studies analysed anastomotic technique. This might be because anastomotic technique is usually only considered relevant in the early postoperative period, with little or no long-term impact on patients.

Nevertheless, postoperative complications do have a clear impact on patients’ QoL. Several RCTs have reported better QoL after colorectal surgery in those patients that had no postoperative complications, mainly after rectal cancer surgery^17–19^. Again, those reports pool both colon and rectal cancer patients in their analysis. No multicentric, prospective studies have been performed to specifically assess the impact of minimally invasive right colectomy on patients’ QoL, or the impact of postoperative complications in this group of patients. If ICA has the potential to decrease postoperative complications, it might also have the potential to improve QoL.

The MIRCAST was developed to analyse the impact of ICA on postoperative complications, IH, QoL, and mid-term oncological outcomes^20^. The results regarding postoperative outcomes were published in 2023^10^, and mid-term outcomes, including IH, QoL, and oncological outcomes are presented in this manuscript.

## Methods

### Study design and setting

MIRCAST is an international, multicentre, prospective, observational, non-randomized, parallel, four-cohort study. The study was performed according to a published protocol^20^ and is supported by the European Society of Coloproctology (ESCP). The study followed the principles of the Declaration of Helsinki and received approval from ethical boards across 59 participating centres in Europe. The study was registered at ClinicalTrials.gov (NCT03650517) in 2018.

Colorectal surgeons from geographical Europe with experience of 30 or more minimally invasive right colectomy procedures per year, working in high-volume institutions, preferably with an enhanced recovery after surgery (ERAS) protocol already implemented, were invited to participate.

A site initiation visit was conducted in all centres before enrolment of the first patient. Data collection was undertaken prospectively within a secure database (Open Clinica, Waltham, MA, USA) from the preoperative and intraoperative assessments, and the 30-day, 90-day, 1-year, and 2-year follow-up. Remote and in-person data monitoring was performed by two clinical research assistants. In-person data monitoring was undertaken for 25% of randomly selected enrolled patients.

Patients were classified into one of four cohorts according to the planned surgical approach, which entailed two treatment assignments: ICA or ECA, and laparoscopic (LAP; using any laparoscopic device) or robotic-assisted surgery (RAS; using any of the available robotic systems at the participant institutions). Different surgeons from the same institution could enrol patients in different cohorts.

### Participants

Inclusion criteria were adult patients aged 18 years or older with a tumour (benign or malignant) in the right colon requiring an elective right colectomy with curative intent, a life expectancy of at least 12 weeks, and adequate performance status (Eastern Cooperative Oncology Group grade 0, 1 or 2). Before inclusion, all patients voluntarily signed and dated an informed consent form.

Exclusion criteria were: cT4b tumours, metastatic disease, planned colonic surgery along with other major concomitant procedures, or inflammatory bowel disease. Patients who were pregnant or suspected to be pregnant, had a co-morbid illness or condition precluding surgery, were undergoing an emergency procedure, or were unwilling to comply with all the follow-up study requirements were also excluded.

### Interventions

Patients were recruited to one of four cohorts depending on the surgeon’s experience and practice: LAP ICA, RAS ICA, LAP ECA, or RAS ECA. A screening log was maintained at each centre to identify potential selection bias. For the ICA cohorts, a Pfannenstiel incision was the chosen wound for specimen extraction. If an operation could not be completed using any of these minimally invasive techniques, the procedure was converted to open surgery.

Secondary outcomes included: 2-year IH rate, 2-year disease free and overall survival, and the EuroQol Five Dimensions (EQ-5D; EuroQol Group, Rotterdam, The Netherlands) and European Organization for Research and Treatment of Cancer quality-of-life (EORTC QoL) core questionnaire C30 (a 30-item questionnaire meant to assess QoL of cancer patients) and CR29 (a colorectal cancer–specific module meant to assess QoL of colorectal cancer patients).

### Statistical analysis

Quantitative variables were described using central tendency and dispersion measures (arithmetic mean and standard deviation), whereas qualitative variables were analysed through absolute and relative frequencies. Associations between qualitative variables were tested using the chi-square test or Fisher’s exact test, as appropriate. Logistic regression and odds ratios were used to model relationships between dichotomous dependent variables and independent variables, following normality checks with the Kolmogorov–Smirnov test. Mean differences were compared using Student’s *t*-test for normally distributed variables or the Mann–Whitney U test otherwise.

Propensity score adjustments using multinomial regression were explored for secondary outcomes, considering potential confounders such as age, sex, BMI, ASA classification, Charlson Comorbidity Index (CCI), previous abdominal surgery, previous abdominal disease, bowel preparation, and preoperative antibiotics. As these factors were not significant in the model, analyses proceeded without propensity score adjustments.

Adjustments were made for interactions between ICA and RAS as explanatory variables when used individually. No adjustments were necessary when combining ICA and RAS as a single variable.

**Fig. 1:**
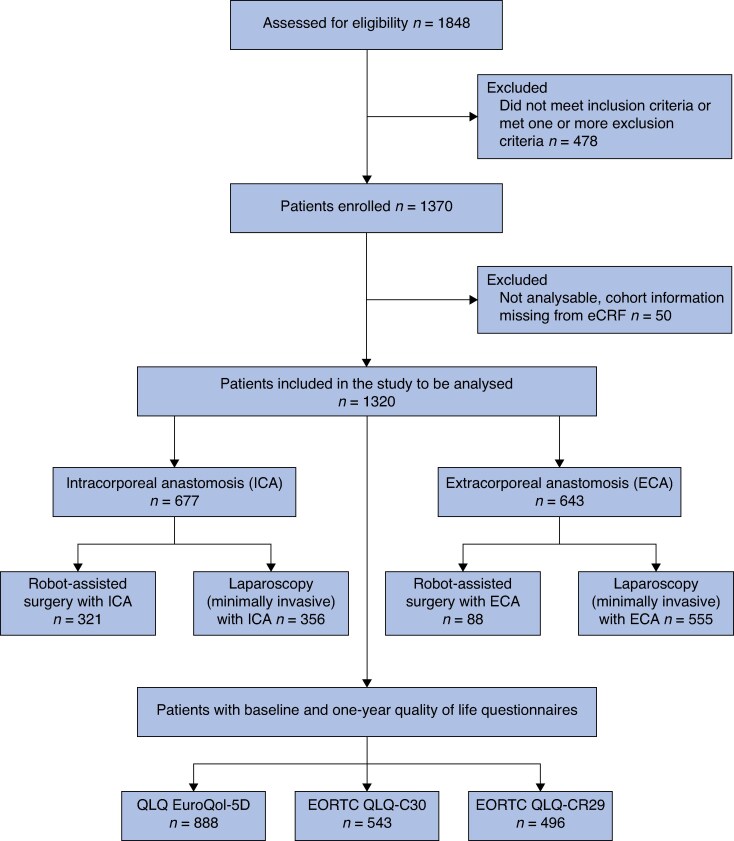
MIRCAST study flow chart

For the analysis of quality of life, patients with data recorded in the baseline questionnaire and the 1-year follow-up questionnaire were analysed (*Fig. 1*). The analysis of EQ-5D, CR29, and C30 questionnaires was undertaken using their respective user guides^21–24^.

eCRF, electronic case record form; ICA, intracorporeal anastomosis; ECA, extracorporeal anastomosis; EORTC, European Organization for Research and Treatment of Cancer; QLQ, quality of life questionnaire.

Oncological outcomes were only assessed for patients with colon cancer in the pathological report. Survival probabilities were assessed using Kaplan–Meier curves, with log-rank tests comparing groups.

Patients were analysed on an intention-to-treat basis, excluding those with missing data. Statistical significance was set at *P* < 0.05, and all analyses were conducted using Stata® 15 software (StataCorp, USA).

## Results

As previously reported, 1848 patients were assessed for eligibility between 2018 and 2021^10^. After reviewing the inclusion and exclusion criteria and available information on the cohort, 1320 patients were included in the study (643 patients in the ECA cohort and 677 in ICA; 555 in LAP ECA, 356 in LAP ICA, 88 in RAS ECA, and 321 in RAS ICA) (*Fig. 1*).

### Incisional hernia

The overall rate of IH at 2 years was 3.48% (46/1320 patients; *Table 1*). Of those, 37% (17/46) underwent a surgical repair during the two years of follow-up. ICA significantly reduced the risk of hernia compared to ECA (OR 0.21, 95% c.i.: 0.09 to 0.43, *P* < 0.001). RAS independently reduced the likelihood of hernia compared to LAP (OR 0.05, 95% c.i.: 0.006 to 0.33, *P* = 0.002). In cohort analysis, the RAS ICA group showed a significantly lower hernia rate compared to the LAP ECA group (OR 24.69, 95% c.i.: 3.36 to 181.06, *P* = 0.002), whereas the comparison between RAS ICA and LAP ICA showed a non-significant trend (OR 7.20, 95% c.i.: 0.89 to 58.9, *P* = 0.63). Conversely, the analysis between LAP ICA and LAP ECA revealed a significantly higher incidence of hernia in LAP ECA (OR 3.43, 95% c.i.: 1.57 to 7.47, *P* = 0.002). Analysis of the chosen site for specimen extraction revealed that most hernias were related to midline and subcostal transvers incisions, most commonly utilized during ECA (96%), and LAP (60.17%) (*Table 1*).

**Table 1:** Descriptive analysis of specimen extraction site, postoperative complications, incisional hernia, local recurrence and metastatic disease

		Missing data (%)	Entire population n (%)	ICA—ECA n (%)		RAS—LAP n (%)	
				ICA 1	ECA 0	RAS 1	LAP 0
Specimen extraction site	Pfannenstiel	11.59	566 (48.5%)	544 (88.0%)	22 (4.0%)	245 (67.9%)	321 (39.8%)
	T + M		601 (51.5%)	74 (11.9%)	527 (96%)	116 (32.1%)	485 (60.2%)
Presence of complications	No	0	980 (73.2%)	512 (75.7%)	468 (72.8%)	326 (79.7%)	654 (71.8%)
	Yes		340 (26.8%)	165 (24.3%)	175 (27.2%)	83 (20.3%)	257 (28.2%)
Incisional hernia	No	0	1274 (96.5%)	668 (98.7%)	606 (94.3%)	408 (99.8%)	866 (95.1%)
	Yes		46 (3.5%)	9 (1.3%)	37 (5.7%)	1 (0.2%)	45 (4.9%)
Local recurrence	No	17.35	1070 (98.2%)	527 (97.4%)	543 (98.7%)	340 (97.9%)	730 (98.1%)
	Yes		21 (1.8%)	14 (2.6%)	7 (1.3%)	7 (2.0%)	14 (1.9%)
Metastatic disease	No	17.35	1026 (94.0%)	512 (94.6%)	514 (93.5%)	328 (94.5%)	698 (93.8%)
	Yes		65 (5.9%)	29 (5.4%)	36 (6.5%)	19 (5.5%)	46 (6.2%)

### Oncological outcomes

The overall rate of local recurrence at 2 years was 1.83% (20/1091 patients; *Table 1*). No significant differences were found in local recurrence between ICA and ECA (χ^2^(1) = 2.04, *P* = 0.15), or RAS and LAP (χ^2^(1) = 0.016, *P* = 0.89). The overall rate of metastatic disease at 2 years was 5.9% (65/1091 patients; *Table 1*). The analyses did not reveal significant differences when comparing ICA and ECA (χ^2^(1) = 0.56, *P* = 0.45) or RAS and LAP (χ^2^(1) = 0.11, *P* = 0.74). Two-year overall survival (OS) was 95.2%, and disease-free survival (DFS) was 95.1%. OS and DFS per pathological stage were respectively: Stage I 98.7% and 98.7%, Stage II 97.2% and 97.2%, Stage III 91.8% and 91.6%, and Stage IV 85.5% and 65.2%. No difference in OS was observed when comparing ICA and ECA (χ^2^(1) = 0.54, *P* = 0.46) or RAS and LAP (χ^2^(1) = 0.07, *P* = 0.79).

#### Quality of life

EQ-5D was available for analysis in 888 patients, C30 in 543 patients, and CR29 in 496 patients (*Fig. 1*).

##### EQ-5D

Analysis of the EQ-5D questionnaire showed a significant improvement in QoL of the population (888 patients) one year after surgery (coefficient 0.04, 95% c.i.: 0.02 to 0.06, *P* < 0.001). There is a significant difference in the probability of increasing QoL one year after surgery between the ECA and ICA groups (OR 1.33, 95% c.i.: 1.02 to 1.75, *P* = 0.038). No significant differences were found between RAS and LAP (OR 0.96, 95% c.i.: 0.72 to 1.29, *P* = 0.79). Considering the specimen extraction site (transverse and midline *versus* Pfannenstiel), significant differences are observed with improved QoL associated with a Pfannenstiel incision (OR 0.9, 95% c.i.: 0.82 to 0.99, *P* = 0.036; *Table 2*).

**Table 2:** Quality of life (EQ5D) one year after surgery compared to baseline

	OR	Std. error	z	*P* > IzI	95% c.i.	
ICA *versus* ECA	1.33	0.18	2.08	0.038	1.02	1.75
RAS *versus* LAP	0.96	0.14	–0.27	0.788	0.72	1.29
Pfannenstiel *versus* transverse/midline	0.90	0.04	–0.10	0.036	0.82	0.99
Complications	0.94	0.14	–0.42	0.677	0.69	1.26
Incisional hernia	0.94	0.32	–0.19	0.851	0.48	1.83
Local recurrence	1.60	0.83	0.89	0.372	0.57	4.44
Metastasis	1.11	0.31	0.37	0.711	0.64	1.93

No significant differences were observed when repeating the analysis considering the presence or absence of complications (OR 0.94, 95% c.i.: 0.70 to 1.26, *P* = 0.68), IH (OR 0.93, 95% c.i.: 0.48 to 1.83, *P* = 0.85), local recurrence (OR 1.59, 95% c.i.: 0.57 to 4.44, *P* = 0.37), or metastatic disease (OR 1.11, 95% c.i.: 0.64 to 1.93, *P* = 0.71) (*Table 2*).

##### C30 and CR29

C30 and CR29 questionnaires revealed a significant improvement in QoL one year after surgery compared with baseline; C30 coefficient 1.73 (95% c.i.: 1.37 to 2.08, *P* < 0.001), and CR29 coefficient 2.43 (95% c.i.: 1.90 to 2.97, *P* < 0.001). ICA showed significant improvement in QoL over ECA one year after surgery in both questionnaires; C30 OR 1.67 (95% c.i.: 1.18 to 2.37, *P* = 0.004), and CR29 OR 1.84 (95% c.i.: 1.28 to 2.63, *P* = 0.001). RAS demonstrated a significant improvement in QoL one year after surgery in CR29 when compared with LAP (OR 1.77, 95% c.i.: 1.2 to 2.6, *P* = 0.004), with no significant improvement in C30 (OR 0.97, 95% c.i.: 0.66 to 1.4, *P* = 0.86) (*Table 3*).

**Table 3:** Quality of life (C30 and CR29) one year after surgery compared to baseline in the study population and subgroup with postoperative complications

QLQ		OR	Std. error	z	*P* > IzI	95% c.i.	
**Overall population**							
C30	ICA *versus* ECA	1.67	0.29	2.91	0.004	1.18	2.37
	RAS *versus* LAP	0.97	0.19	−0.18	0.857	0.66	1.41
CR29	ICA *versus* ECA	1.84	0.34	3.31	0.001	1.28	2.63
	RAS *versus* LAP	1.77	0.35	2.91	0.004	1.20	2.59
**With postoperative complications**							
C30	ICA *versus* ECA	2.03	0.66	2.17	0.030	1.07	3.83
	RAS *versus* LAP	1.45	0.53	1	0.32	0.69	3.0
CR29	ICA *versus* ECA	2.75	0.95	2.93	0.003	1.4	5.4
	RAS *versus* LAP	3.95	1.62	3.35	0.001	1.76	8.84

In a subgroup analysis of patients with postoperative complications, ICA showed significant improvement in QoL over ECA one year after surgery in both EORTC questionnaires; C30 OR 2.03 (95% c.i.: 1.07 to 3.83, *P* = 0.03), and CR29 OR 2.75 (95% c.i.: 1.4 to 5.4, *P* = 0.003). RAS demonstrated a significant improvement in QoL (CR29) one year after surgery in patients with postoperative complications when compared with LAP (OR 3.95, 95% c.i.: 1.76 to 8.84, *P* = 0.001), with no significant improvement in C30 (OR 1.45, 95% c.i.: 0.7 to 3.0, *P* = 0.32) (*Table 3*).

Significant differences were also found when analysing the C30 Global Health Status in the presence or absence of complications, with patients without complications showing a higher QoL (OR 0.68, 95% c.i.: 0.50 to 0.93, *P* = 0.0.014). Considering the specimen extraction site (transverse and midline *versus* Pfannenstiel), significant differences were observed with an improved QoL associated with use of a Pfannenstiel incision (OR 0.85, 95% c.i.: 0.77 to 0.94, *P* = 0.002). No significant differences were observed when repeating the analysis considering the presence of IH (OR 1.55, 95% c.i.: 0.66 to 3.68, *P* = 0.315), local recurrence (OR 1.05, 95% c.i.: 0.36 to 3.07, *P* = 0.923), or metastatic disease (OR 1.46, 95% c.i.: 0.76 to 2.79, *P* = 0.248) (*Table 4*).

**Table 4:** Quality of life (C30—Global Health Status) one year after surgery compared to baseline

	OR	Std. error	z	*P* > IzI	95% c.i.	
Pfannenstiel *versus* transverse/midline	0.85	0.04	–3.12	0.002	0.77	0.94
Complications	0.68	0.11	–2.45	0.014	0.49	0.93
Incisional hernia	1.55	0.68	1.00	0.315	0.66	3.68
Local recurrence	1.05	0.58	0.10	0.923	0.36	3.07
Metastasis	1.46	0.48	1.16	0.248	0.77	2.79

## Discussion

The MIRCAST study group, involving 59 institutions and more than 100 surgeons across Europe, has established the largest prospective, non-randomized, monitored, multicentre cohort study focusing on intracorporeal anastomosis (ICA) after minimally invasive right hemicolectomy to date. It is also the first study investigating QoL in patients after right hemicolectomy with different anastomotic techniques (ICA and ECA) and surgical approaches (LAP and RAS).

The study’s observational design, variation in enrolments between the cohorts, and missing QoL data for some patients are some of the limitations of our study. Restricting inclusion from surgeons of high-volume centres could also limit the generalizability. Low enrolment in the RAS ECA cohort may have had an impact on the comparison between LAP and RAS. Although more than 800 QoL questionnaires were collected and analysed, missing data might have generated some bias.

A higher incidence of IH after ECA with midline or transverse incisions for specimen extraction has been reported in several publications, when compared with ICA or Pfannenstiel incision^13,25,26^. It is well known that IH has a negative impact on quality of life after colonic cancer resections^27,28^, with a 5-year recurrence rate of at least 40%^29^. In our study, the rate of IH was significantly lower in the ICA group compared to the ECA group (1.3% *versus* 5.7% respectively). Similarly, RAS showed a protective effect against IH, with a 92.9% reduction in likelihood compared to LAP.

This study did not find any significant differences between ICA and ECA, or between RAS and LAP, in respect of oncological outcomes at two years. Patients had a high (over 90%) OS and DFS, which might be explained by the early stage of the tumours treated in this trial (91% T1–T3 and only 23% N+). It must be noted that T4b and metastatic disease at screening were exclusion criteria, and that patients were enrolled in highly specialized centres. As previously reported, RAS was associated with a greater number of harvested lymph nodes (OR 3.93, *P* < 0.001), but this had no impact on local recurrence or metastatic disease in our study. We conclude therefore that choice of anastomotic technique has no independent effect of oncological outcomes.

Multiple QoL questionnaires were used to assess different QoL domains in detail. EQ-5D is a generic health-related questionnaire, whereas EORTC QLQ-C30 is a cancer specific questionnaire designed for clinical trials, and EORTC QLQ-CR29 is a colorectal cancer–specific module. EQ-5D revealed a significant improvement in overall quality of life one year after surgery, with ICA showing advantages over ECA in certain domains. These differences did not always reach statistical significance. Pfannenstiel incision, a surrogate marker of ICA, was associated with a significant increase in QoL one year after surgery when compared with other specimen extraction sites. Although ICA showed a clear improvement in all QoL questionnaires when compared with ECA, RAS only showed an improvement in the colorectal cancer specific questionnaire (CR29) when compared with LAP.

It is well known that postoperative complications, and specifically anastomotic leakage, have an impact on QoL after colorectal surgery^17–18^. MIRCAST mirrors this outcome, showing a significant impact of postoperative complications on QoL (based on C30 global health analysis). The degree to which postoperative complications have an impact on QoL might be modified by using different surgical techniques. Subgroup analysis of CR29 and C30 questionnaires demonstrated a benefit of ICA in QoL one year after surgery in those patients that had postoperative complications, when compared to ECA (C30 OR 2.03, *P* = 0.03; CR29 OR 2.75, *P* = 0.003). In the same subgroup analysis, RAS outperformed LAP, showing a significant improvement one year after surgery in the CR29 questionnaire (OR 3.95, *P* = 0.001).

As reported in our previous manuscript, ICA outperformed ECA in reducing overall postoperative complications (ICA *versus* ECA 24% *versus* 27%, *P* = 0.001, and RAS ICA *versus* LAP ECA 18.9% *versus* 27.5%, *P* = 0.005 respectively), and our novel data show that it is also associated with a lower incidence of IH. It can be a challenge to understand why choice of anastomotic technique may have an impact on QoL, but based on the data presented in this study, we suggest that ICA improves QoL by decreasing rates of both postoperative complications and IH. The advantages observed in this study lead us to recommend the use of ICA as a standard of care.

## Collaborators

Juan García Cardo MD, Grupo de Investigación e Innovación en Cirugía, IDIVAL and Colorectal Surgery Unit, Marqués de Valdecilla University Hospital, Santander, Spain; Marcos Gómez Ruiz MD, PhD, Grupo de Investigación e Innovación en Cirugía, IDIVAL, Colorectal Surgery Unit, Marqués de Valdecilla University Hospital, Santander, Spain; Eloy Espin Basany MD, Colorectal Surgery Unit, Vall d’Hebrón University Hospital, Barcelona, Spain; Mindaugas Tiskus MD, Hospital of Southern Denmark, Aabenraa, Denmark; Ugo Pace MD, Istituto Nazionale Tumori—IRCSS—Fondazione Pascale, Naples, Italy; Tarja Pinta MD, PhD, Seinäjoki Central Hospital, Seinäjoki, Finland; Paolo Pietro Bianchi MD, General Surgery Unit, Department of Health Sciences and DISS, University of Milan, San Paolo Hospital, Milan, Italy; Andrea Coratti MD, Department of General and Emergency Surgery and Digestive Diseases—Misericordia Hospital of Grosseto, Italy; Roberto Persiani MD, Policlínico Agostino Gemelli, Rome, Italy; Roberto Coppola MD, Campus Biomedico di Roma, Rome, Italy; Nuria Truan Alonso MD, Hospital Universitario Central de Asturias, Oviedo, Spain; Franco Marinello MD, Hospital Vall d’Hebron, Barcelona, Spain; Miquel Kraft Carre MD, Hospital Vall d’Hebron, Barcelona, Spain; Mirjana Komljen MD, Hospital of Southern; Denmark, Aabenraa, Denmark; Shadi Andos MD, Hospital of Southern Denmark, Aabenraa, Denmark; Paolo Delrio MD, Istituto Nazionale Tumori—IRCSS—Fondazione Pascale, Naples, Italy; Daniela Rega MD, Istituto Nazionale Tumori—IRCSS—Fondazione Pascale, Naples, Italy; Giuseppe Giuliani MD, Ospedale La Misericordia, Grosseto, Italy; Lucia Salvischiani MD, Ospedale La Misericordia, Grosseto, Italy; Alberto Biondi MD, Policlinico Agostino Gemelli, Rome, Italy; Laura Lorenzon MD, Policlinico Agostino Gemelli, Rome, Italy; Damiano Caputo MD, Campus Biomedico di Roma, Rome, Italy; Vincenzo La Vaccara MD, Campus Biomedico di Roma, Rome, Italy; Daniel Fernández Martinez MD, Hospital Universitario Central de Asturias, Asturias, Spain; Carmen Cagigas Fernández MD, PhD, Grupo de Investigación e Innovación en Cirugía, IDIVAL, Colorectal Surgery Unit, Marqués de Valdecilla University Hospital, Santander, Spain; Lidia Cristobal Poch MD, PhD, Grupo de Investigación e Innovación en Cirugía, IDIVAL, Colorectal Surgery Unit, Marqués de Valdecilla University Hospital, Santander, Spain; Gina Lladó-Jordan PhD, Grupo de Investigación e Innovación en Cirugía, IDIVAL, Santander, Spain; Camilo Palazuelos Calderón PhD, Grupo de Investigación e Innovación en Cirugía, IDIVAL, Santander, Spain; Lucía Lavín Alconero PhD, Unidad de Ensayos Clínicos IDIVAL, Santander, Spain; Vincenzo Vigorita MD, Hospital Álvaro Cunqueiro, Vigo, Spain; Raquel Sánchez Santos MD, Hospital Álvaro Cunqueiro, Vigo, Spain; Paula Fernández Rodriguez MD, Hospital Álvaro Cunqueiro, Vigo, Spain; Fernando Jimenez Escobar MD, Hospital Galdakao Usansolo, Galdakao, Spain; Tamara Fernández Miguel MD, Hospital Galdakao Usansolo, Galdakao, Spain; Vicente Portugal Porras MD, Hospital Galdakao Usansolo, Galdakao, Spain; Alejandro Romero de Diego MD, Hospital Clínico Universitario de Valladolid, Valladolid, Spain; Maria Ruíz Soriano MD, Hospital Clínico Universitario de Valladolid, Valladolid, Spain; Beatriz de Andrés Asenjo MD, Hospital Clínico Universitario de Valladolid, Valladolid, Spain; Dursun Bugra MD, Koç University, Istanbul, Turkey; Emre Balik MD, Koç University, Istanbul, Turkey; Emre Özoran MD, Koç University, Istanbul, Turkey; Andrea Muratore MD, Ospedale ‘Edoardo Agnelli’ Pinerolo, Pinerolo, Italy; Marcello Calabrò MD, Ospedale ‘Edoardo Agnelli’ Pinerolo, Pinerolo, Italy; Antonio La Terra MD, Ospedale ‘Edoardo Agnelli’ Pinerolo, Pinerolo, Italy; Ángel Reina Duarte MD, H.U. Torrecárdenas, Almería, Spain; Pälvi Vento MD, Kymenlaakso Central Hospital, Kotka, Finland; Inna Lupina MD, Kymenlaakso Central Hospital, Kotka, Finland; David Moro-Valdezate MD, Hospital Clínico Universitario de Valencia, Valencia, Spain; José Martín-Arévalo MD, Hospital Clínico Universitario de Valencia, Valencia, Spain; Juan Ocaña Jiménez MD, Hospital Ramón y Cajal, Madrid, Spain; Araceli Ballestero—Pérez MD, Hospital Ramón y Cajal, Madrid, Spain; Ellen Van Eetvelde MD, UZ Brussel, Brussels, Belgium; Daniel Jacobs-Tulleneers-Thevissen MD, UZ Brussel, Brussels, Belgium; Irshad Shaikh MD, Norfolk and Norwich University Hospital, Norwich, UK; Dolly Dowsett MD, Norfolk and Norwich University Hospital, Norwich, UK; Esther Kreisler Moreno MD, Hospital Universitario Bellvitge, Barcelona, Spain; Ana Gálvez Saldaña MD, Hospital Universitario Bellvitge, Barcelona, Spain; Antonino Spinelli MD, Humanitas Research Hospital, Milan, Italy; Francesca Di Candido MD, Humanitas Research Hospital, Milan, Italy; Luis Miguel Jimenez Gomez MD, Hospital General Universitario Gregorio Marañón, Madrid, Spain; Elena Hurtado Caballero MD, Hospital General Universitario Gregorio Marañón, Madrid, Spain; Andreas Türler MD, Johanniter KH Bonn, Bonn, Germany; Anna Krappitz MD, Johanniter KH Bonn, Bonn, Germany; Luca Morelli MD, SD Chirurgia Generale Universitaria, Pisa, Italy; Annalisa Comandatore MD, SD Chirurgia Generale Universitaria, Pisa, Italy; Matteo Palmeri MD, SD Chirurgia Generale Universitaria, Pisa, Italy; Vicente Simó MD, Complejo Asistencial Universitario de León, León, Spain; Jorge Arredondo Chaves MD, Complejo Asistencial Universitario de León, León, Spain; Benno Mann MD, Augusta Krankenhaus Bochum, Bochum, Germany; Gintautas Virakas MD, Augusta Krankenhaus Bochum, Bochum, Germany; Jim Khan MD, Portsmouth Hospital NHS Trust, Portsmouth, UK; Ismail Gögenur MD, Zealand University Hospital, Denmark; Niclas Dohrn MD, Zealand University Hospital, Denmark; Eduardo Ferrero Herrero MD, Hospital 12 de Octubre, Madrid, Spain; Eduardo Rubio González MD, Hospital 12 de Octubre, Madrid, Spain; Javier Sanchez Gonzalez MD, Hospital Universitario Río Hortega de Valladolid, Valladolid, Spain; Ekta Choolani Bhojwani MD, Hospital Universitario Río Hortega de Valladolid, Valladolid, Spain; Francesk Mulita MD, General University Hospital of Patras, Patras, Greece; Vasileios Leivaditis MD, General University Hospital of Patras, Patras, Greece; Goran Šantak MD, County Hospital Požega, Požega, Croatia; Matteo Frasson MD, Hospital La Fe, Valencia, Spain; Marta Nieto MD, Hospital La Fe, Valencia, Spain; Jakob Lykke MD, Herlev University Hospital, Denmark; Niclas Dohrn MD, Herlev University Hospital, Denmark; Mauro Garino MD, Rivoli Hospital, Turin, Italy; Chiara Marafante MD, Rivoli Hospital, Turin, Italy; Antonio Arroyo MD, Hospital General Universitario de Elche, Elche, Spain; Cristina Lillo-García MD, Hospital General Universitario de Elche, Elche, Spain; Carlos Placer Galan MD, Hospital Universitario Donostia, Donostia, Spain; José María Enriquez Navascués MD, Hospital Universitario Donostia, Donostia, Spain; Wanda Luisa Petz MD, IEO—European Institute of Oncology, Milan, Italy; Simona Borin MD, IEO—European Institute of Oncology, Milan, Italy; Philippe Rouanet MD, ICM—Institut Régional du Cancer de Montpellier, Montpellier, France; Christophe Taoum MD, ICM—Institut Régional du Cancer de Montpellier, Montpellier, France; Alain Valverde MD, Hôpital de la Croix Saint Simon, Paris, France; Markus Winny MD, Medizinische Hochschule Hannover, Hannover, Germany; Çağrı Büyükkasap MD, Gazi University Faculty of Medicine, Ankara, Turkey; Benoit Romain MD, Strasbourg—Hôpital Hautepierre, Strasbourg, France; Orestis Ioannidis MD, George Papanikolau, Greece; Giuseppe Spinoglio MD, European Oncology Institute, Milan, Italy; David Jayne MD, Department of Colorectal Surgery, University of Leeds, Leeds, UK; Roger Gerjy MD, Mediclinic Middle East, UAE; Sanjay Chaudhri MD, University Hospitals of Leicester NHS Trust, Leicester, UK; Luis Sánchez-Guillén MD, Hospital General Universitario de Elche, Elche, Spain; Alexis Ulrich MD, Lukas Hospital, Neuss, Germany; Tero Rautio MD, Oulu University Hospital, Oulu, Finland; Jesus Bollo Rodriguez MD, Hospital de la Santa Creu i Sant Pau, Barcelona, Spain; Nuno Rama MD, Centro Hospitalar de Leiria, Leiria, Portugal; Federico Perna MD, AOU-Careggi, Florence, Italy; Eric Rullier MD, Hôpital Haut-Lévèque-CHU, Bordeaux, France; Fernando Mendoza MD, Hospital Universitario Príncipe de Asturias, Alcalá de Henares, Spain; Thalia Petropoulou MD, Eurokliniki Athinou, Athens, Greece; Arto Turunen MD, Kanta-Hämeen Keskussairaala, Hämeenlinna, Finland; Mauricio García Alonso MD, H.U. Clínico San Carlos, Madrid, Spain; Anne Mattila MD, Central Finland Central Hospital, Jyväskylä, Finland; Julian Hance MD, St James University Hospital, Leeds, UK; Bertrand Trilling MD, Hôpital Nord CHU Grenoble, La Tronche, France; Imma Prós Ribas MD, Fundació Hospital Sant Joan de Deu de Martorell, Martorell, Spain; Adeline Germain MD, CHRU Nancy Brabois, Vandoeuvre Les Nancy, France; Kai Leong MD, University Hospitals Coventry & Warwickshire NHS Trust, Coventry, UK.

## Data Availability

Marcos Gómez Ruiz, Gina Lladó Jordan, and Camilo Palazuelos Calderón had full access to all the data in the study and take responsibility for the integrity of the data and the accuracy of the data analysis.
